# P-724. The epidemiology, phenotype, and phylogeny of an influenza A/H3N2 virus outbreak among vaccinated U.S. Navy midshipmen

**DOI:** 10.1093/ofid/ofae631.920

**Published:** 2025-01-29

**Authors:** Simon Pollett, Emily Hone, Stephanie A Richard, Kat Schmidt, Mark P Simons, Michele Wayman, Rezalina Tant, Jennifer Rothenberg, Vivian Hogan, Robert O’Connell, Timothy Burgess, Anthony C Fries, Drake Tilley, Rhonda E Colombo

**Affiliations:** Infectious Disease Clinical Research Program, Department of Preventive Medicine and Biostatistics, Uniformed Services University of the Health Sciences, Bethesda, MD, USA, Bethesda, Maryland; Infectious Disease Clinical Research Program, USUHS, Bethesda, Maryland; Infectious Disease Clinical Research Program, Department of Preventive Medicine and Biostatistics, Uniformed Services University of the Health Sciences, Bethesda, MD, USA, Bethesda, Maryland; Infectious Disease Clinical Research Program, USUHS, Bethesda, Maryland; Infectious Disease Clinical Research Program, Department of Preventive Medicine and Biostatistics, Uniformed Services University of the Health Sciences, Bethesda, MD, USA, Bethesda, Maryland; Infectious Disease Clinical Research Program, USUHS, Bethesda, Maryland; Infectious Disease Clinical Research Program, USUHS, Bethesda, Maryland; Infectious Disease Clinical Research Program, USUHS; Henry M. Jackson Foundation for the Advancement of Military Medicine Inc, Bethesda, Maryland; Infectious Disease Clinical Research Program, USUHS; Henry M. Jackson Foundation for the Advancement of Military Medicine Inc, Bethesda, Maryland; Infectious Disease Clinical Research Program, USUHS, Bethesda, Maryland; Infectious Disease Clinical Research Program, Department of Preventive Medicine and Biostatistics, Uniformed Services University of the Health Sciences, Bethesda, MD, USA, Bethesda, Maryland; U.S. Air Force School of Aerospace Medicine, Dayton, Ohio; Naval Health Clinic Annapolis, Annapolis, MD, USA, Annapolis, Maryland; Infectious Disease Clinical Research Program, USUHS; Henry M. Jackson Foundation for the Advancement of Military Medicine, Inc., Bethesda, Maryland

## Abstract

**Background:**

Characterizing influenza outbreaks among vaccinated populations may support optimization of influenza prevention. We used a surveillance study at the United States Naval Academy (USNA) to characterize the epidemiology, phenotype, and phylogeny of a recent influenza A/H3N2 outbreak among vaccinated midshipmen (undergraduate students).

Figure 1

**Figure:**
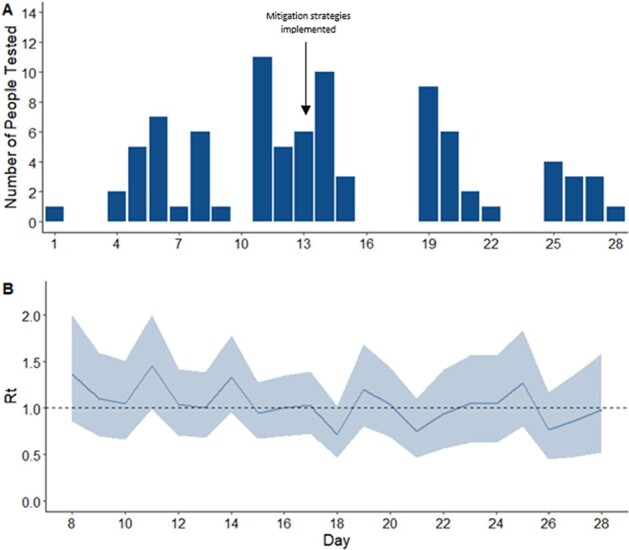

Figure 1A: Influenza A/H3N2 case counts among USNA Midshipmen and Staff Evaluated in February 2024. Day 1 refers to the collection time of the first influenza A/H3N2 outbreak clonal sequence. ‘Mitigation strategies implemented’ included case isolation, case masking, and contact chemoprophylaxis. Figure 1B: Estimated Rt (effective reproductive number) of influenza A/H3N2 virus cases in February 2024. Day 1 refers to the collection timing of first clonal influenza sequence.

**Methods:**

“Acute Respiratory Infections at the Academy” (ARIA) is a study of medically attended ARIs (MAARIs) among USNA midshipmen and staff presenting to the Brigade Medical Unit for clinical care; the USNA comprises ≈ 4000 midshipmen. ARIA abstracts epidemiological, clinical and laboratory data from MAARIs. Residual clinical respiratory tract swabs also undergo multiplex PCR and sequencing of respiratory viruses, including influenza viruses.

**Figure:**
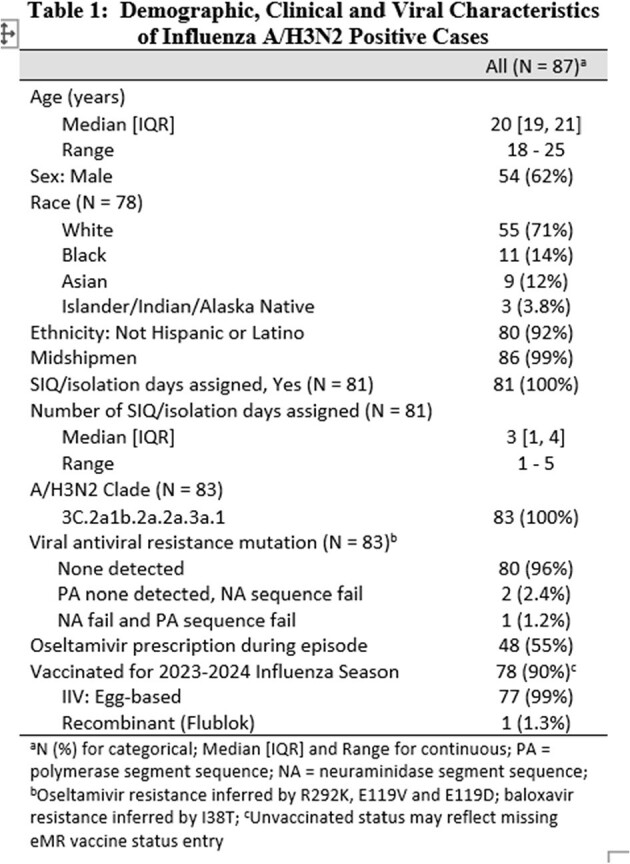

Demographic, Clinical and Viral Characteristics of Influenza A/H3N2 Positive Cases

**Results:**

An outbreak of influenza A/H3N2 emerged in February 2024, with declining cases and R*t* following mitigation strategies (case masking, isolation, and post-exposure oseltamivir; Fig 1). A total of 87 A/H3N2 cases were recorded, predominantly in midshipmen. HA sequence data from 83 cases confirmed local clonal transmission (3C.2a1b.2a.2a.3a.1 clade), consistent with early R*t* estimates (Fig 1, Fig 2). The median age of cases was 20 years and 90% received 2023/2024 Northern Hemisphere influenza vaccine (99% egg-based inactivated influenza vaccine) by October of 2023. Over half (55%) of cases were treated with oseltamivir, and no evidence of antiviral resistance was detected. No cases were hospitalized, and the median sick-in-quarters time was 3 days (Table 1). Phylogenetic analysis did not indicate major genetic divergence from the 2023-2024 Northern Hemisphere vaccine A/H3N2 HA sequence (Fig 3).

**Figure:**
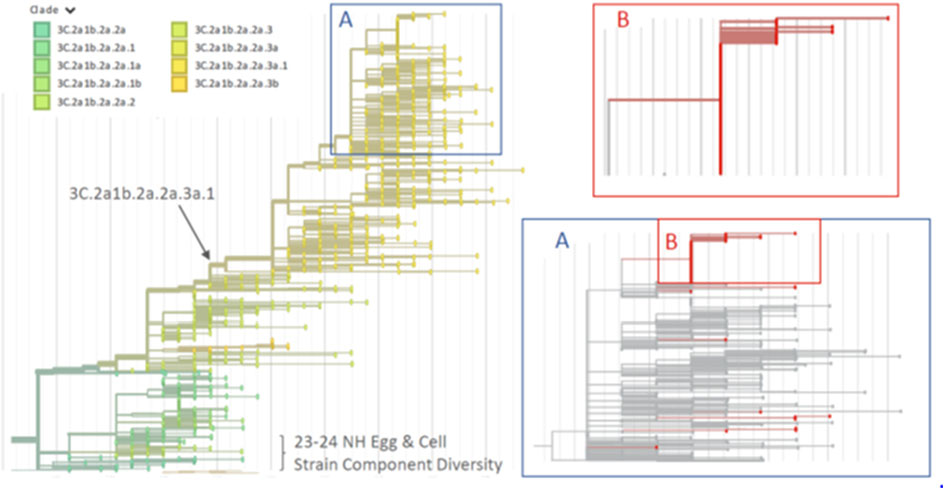

Time-scaled phylogeny of influenza A/H3N2 HA segment genes sequenced from ARIA (red branches) with context background sequences from GISAID. Phylogenetic tree inferred by IQTree and visualized in Auspice using the NextStrain environment. Inset A captures the diversity of 2a.3a.1 influenza cases involved in this outbreak investigation. Inset B shows the largest cluster of cases (n=58) with identical HA gene sequences. The 2023-2024 Northern Hemisphere vaccine strain sequences are indicated. Clade numbers are annotated by color.

**Conclusion:**

Influenza A/H3N2 outbreaks can be impactful in vaccinated congregate populations; further analysis is in progress to identify strain antigenicity markers which may have contributed to this outbreak and predict other outbreaks. Non-pharmaceutical countermeasures and chemoprophylaxis appeared effective in mitigating this outbreak. Our findings highlight the importance of further research into universal influenza vaccines, alternative vaccine dosing regimens, and optimal chemoprophylaxis strategies to prevent such outbreaks.

**Disclosures:**

**Simon Pollett, MBBS**, AstraZeneca: The IDCRP and HJF were funded to conduct an unrelated phase III COVID-19 monoclonal antibody immunoprophylaxis trial as part of US Govt COVID Response **Timothy Burgess, MD, MPH**, AstraZeneca: The IDCRP and HJF were funded to conduct an unrelated phase III COVID-19 monoclonal antibody immunoprophylaxis trial as part of US Govt COVID Response

